# Prescription Department

**Published:** 1897-09

**Authors:** 


TREATMENT OF LARYNGISMUS STRIDULOUS.

℞. Tinct. aconiti.

Tinct. bellad..........aa gtt. x
Aq. laurocerasi........ 15.0
Aq. aurant. flor........ 60.0
Aq. tiliæ.............. 60.0
Syr. simpl.............. 30 0

M. Sig: A teaspoonful every hour. For children two years of age a coffeespoonful is the dose.

Lemoine uses bromide of potash as follows:

℞. Potas. bromat......... 15.0
Syr. chinæ.
Syr. cort. aur..........aa 150 0

M. Sig: Two teaspoonfuls daily for three-year-old child).

During the spasm apply to the anterior surface of the neck a sponge dipped in hot water, as hot as the child will bear it. At the same time steam should be generated in the room, and if mucus has collected in the pharynx, it should be mechanically removed. Look for the cause of these attacks. Frequently adenoid vegetations will be found present in the naso-pharyngeal space and they should be removed.—Pediatrics.

